# A Three-Dimensional Assessment of a Type I Shallow Palatogingival Groove by Cone Beam Computed Tomography: A Case Report

**DOI:** 10.7759/cureus.59835

**Published:** 2024-05-07

**Authors:** Ramachandra Reddy Gowda Venkatesha, Karthik Rajaram Mohan, Saramma Mathew Fenn, Sabitha Gokulraj, Kumar Appusamy

**Affiliations:** 1 Oral Medicine and Radiology, Vinayaka Mission's Sankarachariyar Dental College, Vinayaka Mission's Research Foundation (VMRF-DU), Salem, IND

**Keywords:** bioceramic, mineral tri oxide, selective grinding, cone-beam computed tomography (cbct), palatogingival groove

## Abstract

The palatogingival groove is a developmental anomaly on the palatal surface of the maxillary anterior teeth. The shallow groove, often less than 1 mm, is challenging to diagnose, particularly in radiographic examinations. Such grooves are mistaken for root fractures. In this case study, we explore the prevalence, types, radiological appearances, and treatment options of type I shallow palatogingival grooves encountered in cone beam computed tomography.

## Introduction

The palatogingival groove (PGG) is a developmental aberration that begins as a fissure or invagination in the cingulum of maxillary anterior teeth [1]. The PGG is known by other terms such as a radicular lingual groove or distolingual groove, coronoradicular groove, cinguloradicular groove, vertical developmental radicular groove, developmental radicular anomaly, interruption groove, palatoradicular groove, and embryonic root groove [1]. PGGs commonly occur in maxillary central and lateral incisors. It usually starts in the central fossa area, extends over the cingulum, and continues apically down the root surface. They vary in length as it progresses down toward the apex along the long axis of the root [1].

Ennes and Lara stated that numerous hypotheses have been proposed regarding the genesis of PGGs [2]. The early hypotheses state that PGGs are formed during maxillary growth; an unfavorable position of the maxillary lateral incisor dental germ is encompassed by the canine, premolar, and central incisor, causing the dental germ to fold and create the PGG. In addition to being involved in bone remodeling processes, cellular mediators are produced by mechanotransduction, which converts a physical stimulus into a biological signal. As a result, bone remodeling would continuously occur in response to rising stresses, giving the developing dental germ adequate room. Therefore, it is unlikely that, as previously hypothesized, growth impulses in the maxilla would cause dental germ folding, which is determined by lack of space. In addition, a local mediator causing bone resorption is the epidermal growth factor produced by the epithelial cells of the enamel organ, the cervical loop, and Hertwig's root sheath, which would also ensure sufficient room for the formation of dental germs [2]. The palatogingival etiopathogenesis hypothesis considers the interaction between the dental germ tissues and the molecular expression of their constituent cells. This interpretation of biological events and the similarities of the dental tissue structures observed in both the root developmental groove and the PGG suggests that similar morphogenetic events may be involved in their formation [2]. A modified genetic manifestation influences the PGG formation process [2].

Sometimes, PGGs are misdiagnosed as root fractures [2]. PGG favors plaque buildup, which results in loss of attachment and the progression of periodontal disease [2]. Periapical microsurgery, selective grinding, filling with mineral trioxide, bioceramic, surgical extraction, intentional reimplantation, and guided tissue regeneration are some of the combined endo-perio treatment procedures in the management of the PGG [2].

## Case presentation

**Figure 1:**
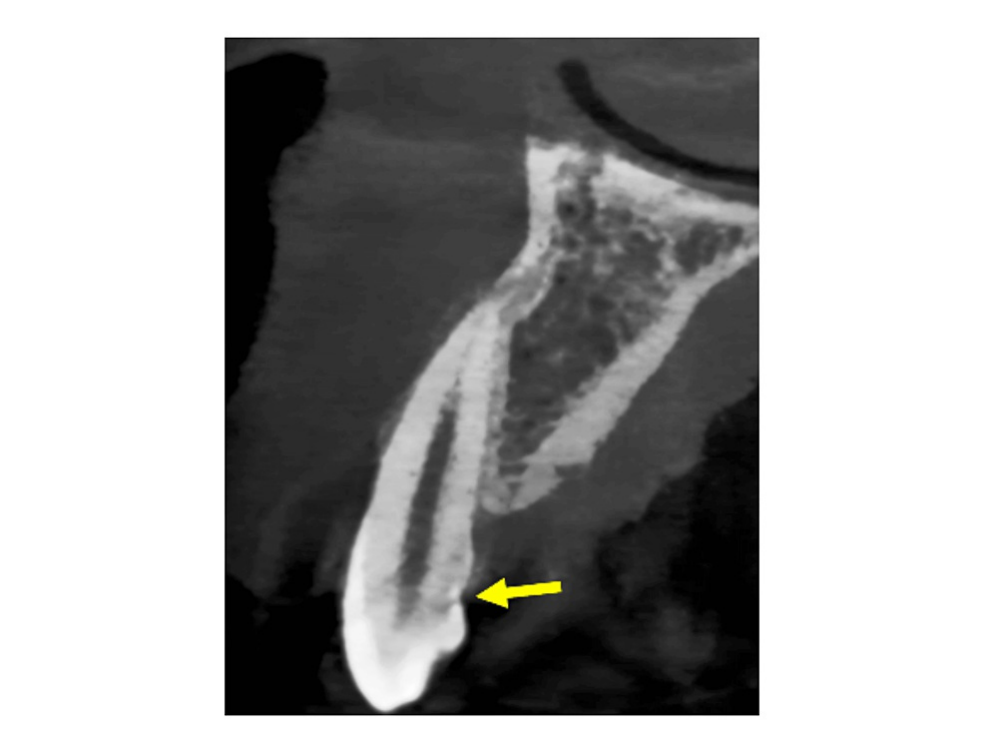
The sagittal section of cone beam computed tomography revealing a horizontal radiolucent line extending on the palatal surface of the right maxillary lateral incisor teeth (yellow arrow).

A 23-year-old female reported a radiological opinion from the periodontia department for a tooth fracture visualized in the sagittal cone beam computed tomography (CBCT) section. The sagittal section CBCT revealed a horizontal radiolucent line extending from the cingulum on the palatal aspect of the maxillary lateral incisor teeth (Figure 1).

**Figure 2:**
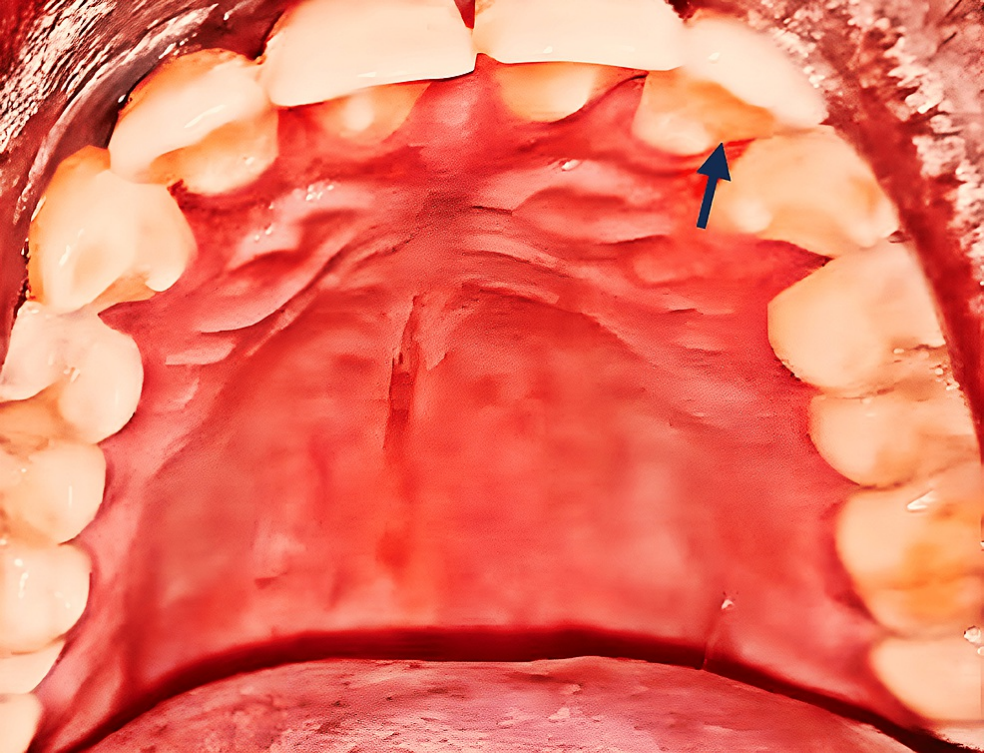
Intraoral examination revealing a palatogingival groove on the palatal surface of the maxillary lateral incisor teeth (blue arrow).

Intraoral examination revealed a groove near the cingulum region on the palatal surface of the maxillary lateral incisor teeth (Figure 2).

**Figure 3:**
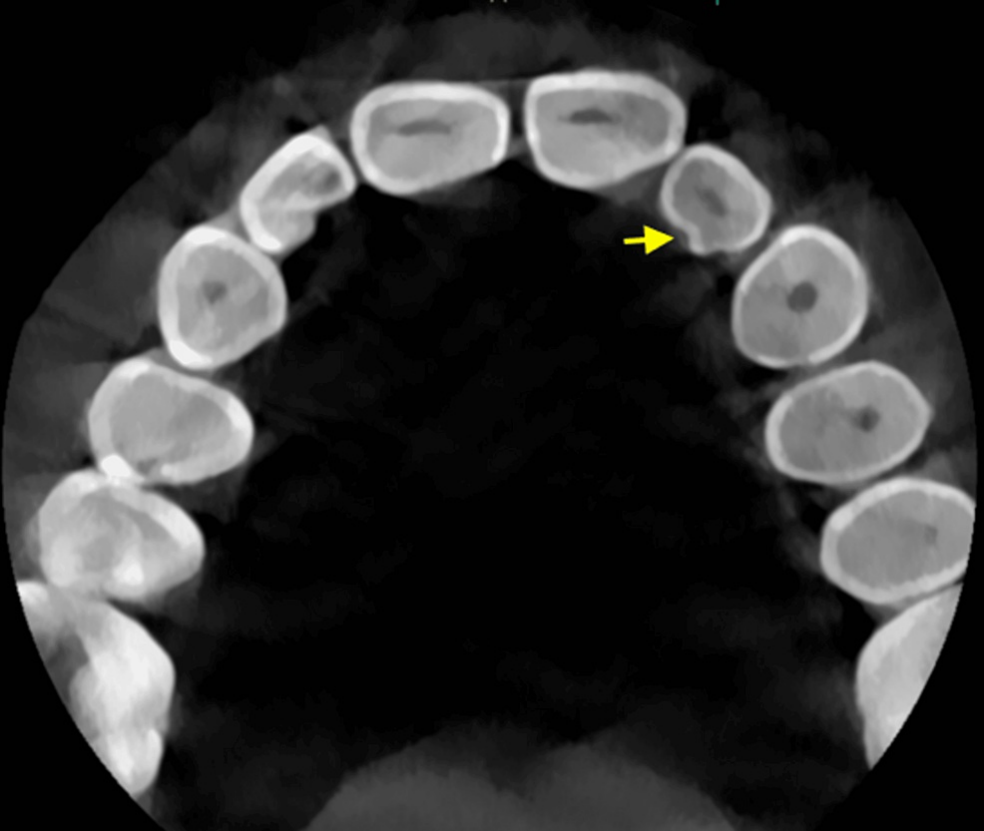
The axial section of cone beam computed tomography revealing palatogingival groove on the palatal surface of the left maxillary lateral incisor teeth.

The axial section of the CBCT, with a 75-micron slice thickness, revealed a depressed layer of enamel groove with an intact outer layer of enamel on the palatal aspect of the crown of the left maxillary lateral incisor teeth (Figure 3).

**Figure 4:**
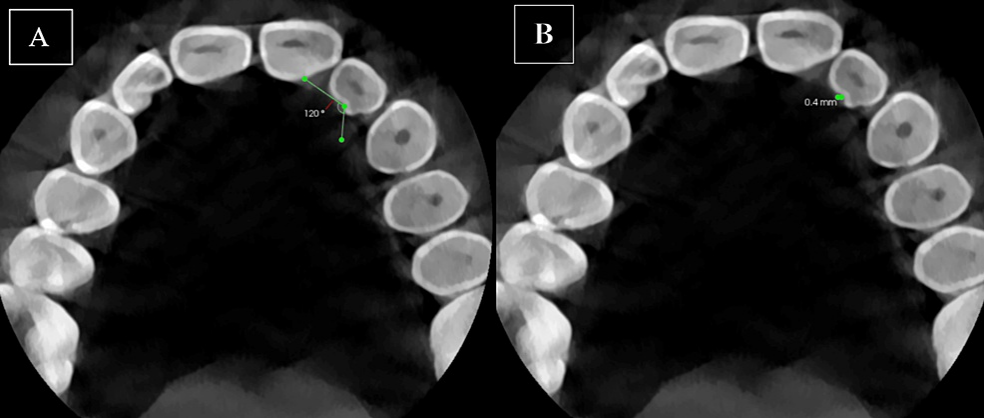
A. The palatogingival groove was at an obtuse angle of 120 degrees. B. The depth of the palatogingival groove was 0.4 mm.

The PGG was at an obtuse angle of 120 degrees and had a depth of 0.4 mm (Figures 4A, 4B).

**Figure 5:**
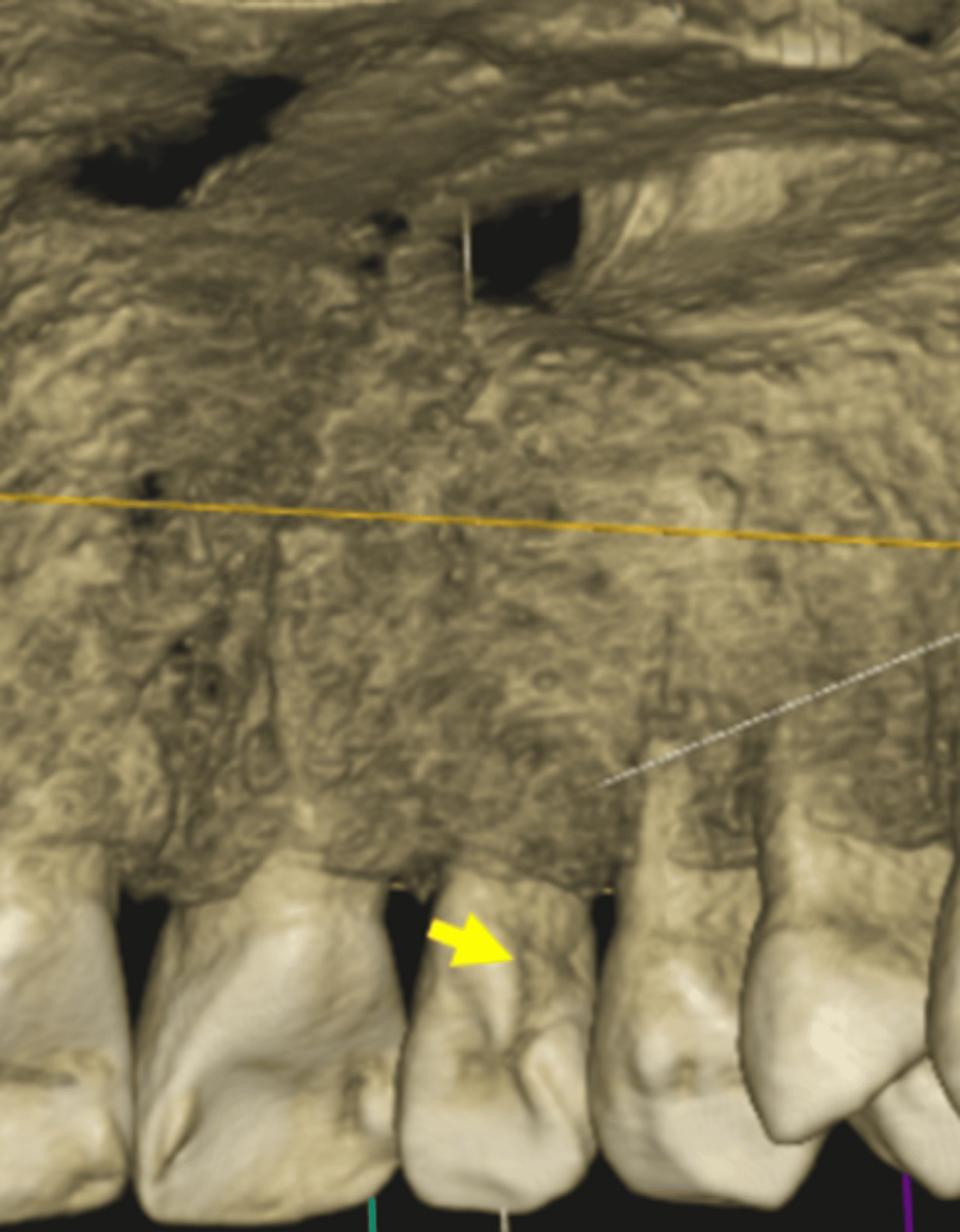
A three-dimensional reconstructed cone beam computed tomography image revealing palatogingival groove on the left maxillary lateral incisor teeth (yellow arrow).

The three-dimensional reconstructed CBCT image also revealed a PGG on the palatal aspect of the left maxillary lateral incisor teeth (Figure 5).

The differential diagnosis for the PGG is crucial, as it aids in differentiating it from other dental conditions such as enamel cracks and dens evaginates. Teeth affected by enamel cracks respond positively to the rubber-wheel test, in which pain occurs when the applied pressure on the rubber wheel is relieved, and such pain does not occur in teeth with PGGs. Similarly, dens evaginates, which commonly affect maxillary premolars, are characterized by an elevated protuberance or lump of enamel. In contrast, the PGG is characterized by a depressed layer of enamel, caused by infolding of inner enamel epithelium and Hertwig's epithelial root sheath during tooth development.

Based on the above clinical and radiographic findings, the PGG showed a depth of 0.4 mm. It did not extend beyond the cementoenamel junction of the crown; thus, we arrived at a final diagnosis of a shallow type I PGG. It is important to note that in this case, no treatment is indicated since the patient is asymptomatic.

Clinical significance

This case highlights the importance of radiological findings of the PGG in CBCT, which can be misdiagnosed as a crown fracture or enamel crack, leading to unwanted interventional surgical procedures.

## Discussion

The PGG is a developmental anomaly of teeth characterized by the presence of a groove on the palatal surface of the maxillary anterior teeth, namely the maxillary central, lateral incisor, and canine. This seemingly innocuous groove can harbor plaque and bacteria, leading to periodontal damage and pulpal pathologic alteration, causing endo-perio lesions. Several factors, including groove type, depth, and location, influence the severity and prognosis of PGG-related diseases. Their small size, funnel-shaped structure, and uneven distribution on the tooth root encourage the adherence of bacteria and plaque, making them potentially vulnerable to periodontal infections. The communication of PGG with the underlying root canal through the accessory canal favors the progression of pulp necrosis, resulting in periapical infections [3].

Aljuailan et al. in their study on 509 CBCT scans representing 2,747 maxillary anterior teeth in Saudi patients reported that 32 patients (6.3%) had PGGs, with a prevalence of 1.3%. Most lateral incisors, 25 (2.77%) contained PGGs. The majority of patients (96.9%) had unilateral PGG, with a higher prevalence in men [4]. PGGs were found in 12 (1.88%) of the 636 front maxillary teeth that were examined (9 [4.2%] lateral incisors, 3 [1.47%] central incisors). Lekshmi et al. in their study among the Indian cohort found the prevalence of PGG in maxillary incisors to be 2.88% [5].

The role of CBCT in diagnosing PGGs cannot be underestimated. This three-dimensional radiographic imaging method plays a crucial role in precisely locating and offering detailed information about the extent of PGGs. The use of CBCT in dental research has significantly contributed to our understanding of PGGs and their implications for dental health [5].

In their retrospective study, Biswas et al. reported the prevalence of PGG in maxillary incisors to be 2.33%. According to the current research, the prevalence of PGGs in the maxillary central and lateral incisors was 5.51% and 1.38%, respectively [6]. A helpful imaging method for determining the location and size of the PGG is CBCT [6]. The classification of the PGG is enumerated in Table 1.

**Table 1:** Classification of palatogingival grooves Table created and modified with permission from Kim et al. [1]

Based on the communication with the root canal	
Simple palatogingival groove	The palatogingival groove does not communicate with the root canal
Complex palatogingival groove	The palatogingival groove communicates with the root canal
Based on the location	
Distal	Distal surface of the teeth
Mesial	Mesial surface of the teeth
Mid-palatal (central)	Mid-palatal (central) surface of the teeth
Based on the groove depth and complexity	
1) Mild	The grooves are gentle depressions of the coronal enamel, which terminate at or immediately after crossing the cementoenamel junction
2) Moderate	The grooves extend to some distance apically along the root surface in the form of a shallow or fissured defect
3) Complex	The grooves are deeply invaginated defects that involve the entire length of the root or that separate an accessory root from the main root trunk
Based on the degree of invagination of the groove toward the pulp cavity	
Shallow/flat	(<1 mm)
Deep	(>1 mm)
Tube-like tunnel	Closed tube that forms a tunnel-like channel
Degree of severity on micro-computed tomography	
Type I	Short groove extending apically not beyond the coronal third of the root and with a normal root canal configuration
Type II	Long shallow groove extending beyond the coronal third of the root and with a normal or simple root canal
Type III	Long deep groove extending beyond the coronal third of the root and with a complex root canal system

The axial section of CBCT images is crucial for visualizing the depth of PGGs. This radiographic method categorizes the PGGs and is the most effective way to determine their depth [4,5]. Radiological examination, particularly with CBCT, is essential for visualizing the PGG and provides more accurate and precise information [5-7].

Anatomical malformations, such as the maxillary lateral incisor's PGG, consistently place the tooth at risk for periodontal and pulpal diseases. CBCT aids in diagnosing, planning treatments, and assessing prognosis by visualizing the morphological changes in teeth with PGGs. The management of PGGs is a complex process that requires a collaborative, interdisciplinary approach. It involves root canal therapy, periodontal therapy, periapical microsurgery, and guided tissue regeneration. This comprehensive approach underscores the complexity and comprehensive nature of managing PGGs [8].

The PGG is removed by preparing and grinding it using a fissure bur and filling with a two-segment restoration technique. This technique involves splitting the cavity in half along the cementoenamel junction. The radicular and coronal portions of the teeth were filled with bioceramics and flowable composite, respectively [9]. Intentional replantation and root excision are viable therapeutic options for managing PGGs in teeth with two roots [10]. A novel combination therapy, such as root conditioning with tetracycline followed by guided tissue regeneration, effectively treats osseous defects caused by PGGs. It has shown significant potential and promising results in arresting disease progression and promoting regeneration [11].

## Conclusions

PGGs are developmental aberrations more common in maxillary central or lateral incisors. The PGG harbors microorganisms and favors plaque build-up, leading to attachment loss and periodontitis. Such grooves also communicate with accessory canals, leading to periapical pathologies even in the absence of dental caries or trauma. Clinicians must recognize the presence of such PGG and appropriately manage it based on their understanding of this condition. This developmental groove is mistaken for a root fracture in CBCT radiographic examination. Hence, thorough knowledge of the radiological findings of such PGGs in CBCT is essential in diagnosing this developmental anomaly and treatment planning.
